# Genetic characterization of intramuscular myxomas

**DOI:** 10.3389/pore.2024.1611553

**Published:** 2024-01-22

**Authors:** William John Hatchett, Marta Brunetti, Kristin Andersen, Maren Randi Tandsæther, Ingvild Lobmaier, Marius Lund-Iversen, Thomas Lien-Dahl, Francesca Micci, Ioannis Panagopoulos

**Affiliations:** ^1^ Section for Cancer Cytogenetics, Institute for Cancer Genetics and Informatics, The Norwegian Radium Hospital, Oslo University Hospital, Oslo, Norway; ^2^ Department of Pathology, The Norwegian Radium Hospital, Oslo University Hospital, Oslo, Norway

**Keywords:** intramuscular myxoma, chromosomal aberrations, GNAS, codon 201, codon 227, Sanger sequencing, Ion torrent

## Abstract

**Introduction:** Intramuscular myxomas are benign tumors that are challenging to diagnose, especially on core needle biopsies. Acquired chromosomal aberrations and pathogenic variants in codon 201 or codon 227 in GNAS complex locus gene (*GNAS*) have been reported in these tumors. Here we present our genetic findings in a series of 22 intramuscular myxomas.

**Materials and methods:** The tumors were investigated for the presence of acquired chromosomal aberrations using G-banding and karyotyping. Pathogenic variants in codon 201 or codon 227 of *GNAS* were assessed using direct cycle Sanger sequencing and Ion AmpliSeq Cancer Hotspot Panel v2 methodologies.

**Results:** Eleven tumors carried chromosomal abnormalities. Six tumors had numerical, four had structural, and one had both numerical and structural chromosomal aberrations. Gains of chromosomes 7 and 8 were the most common abnormalities being found in five and four tumors respectively. Pathogenic variants in *GNAS* were detected in 19 myxomas (86%) with both methodologies. The detected pathogenic variants were p.R201H in nine cases (seven with abnormal and two with normal karyotypes), p.R201C in five cases, all with normal karyotypes, p.R201S in three cases (two with abnormal and one with normal karyotype), p.R201G in one case with a normal karyotype, and p.Q227E in one case with a normal karyotype.

**Conclusion:** Firstly, our data indicate a possible association between chromosomal abnormalities and *GNAS* pathogenic variants in intramuscular myxomas. Secondly, the presence of the rare pathogenic variants R201S, p.R201G and p.Q227E in 26% (5 out of 19) of myxomas with *GNAS* pathogenic variants shows that methodologies designed to detect only the common “hotspot” of p.R201C and p.R201H will give false negative results. Finally, a comparison between Ion AmpliSeq Cancer Hotspot Panel v2 and direct cycle Sanger sequencing showed that direct cycle Sanger sequencing provides a quick, reliable, and relatively cheap method to detect *GNAS* pathogenic variants, matching even the most cutting-edge sequencing methods.

## Introduction

Intramuscular myxomas are rare benign soft tissue tumors, characterized by stellate and/or spindle-shaped cells embedded in a hypo-vascular, abundant myxoid stroma. They were first pathologically described by Enzinginer in 1965 [1] and the description has since been validated in later studies [2–6]. The reported annual incidence rate of intramuscular myxoma is 0.10–0.13/100,000 and mostly occurs in adults, with women having a predisposition for the condition (∼60–70%) [7–9]. Intramuscular myxoma is a somatic disorder with the majority of patients presenting asymptomatically with painless, palpable, well-defined tumors that slowly enlarge to around 2–15 cm in diameter [9]. They are mostly located in the thigh, and do not recur after a simple excision [8]. Multiple intramuscular myxomas are rare, generally occurring as an isolated lesion not associated with other clinical abnormalities [8]. However, multiple intramuscular myxomas in combination with polyostotic fibrous dysplasia can be associated with Mazabraud’s syndrome [7, 9]. Intramuscular myxoma, Mazabraud’s syndrome and the closely related McCune-Albright syndrome (also associated with fibrous dysplasia, café au lait macules and endocrine disorders) are mostly caused by the activating missense mutations in codon 201 or codon 227 of the GNAS complex locus gene (*GNAS*) [10–14].

Pathogenic variants in codon 201 of the *GNAS* gene on chromosome 20 are one of the primary cancer-causing variants in heterotrimeric G proteins and is one of the leading causes of oncogenesis in a variety of low-grade malignant and benign tumors [15]. The *GNAS* gene encodes the stimulatory G-alpha subunit of the heterotrimeric G-protein complex, which regulates the activation of adenylyl cyclase that converts adenosine triphosphate into cyclic adenosine monophosphate. When a cell has a *GNAS* pathogenic variant, there is an overproduction of cyclic adenosine monophosphate and activation of downstream signaling pathways [16–22]. The first description of a somatic *GNAS* pathogenic variant in intramuscular myxomas was published by Okamoto et al. in 2000 [10]. Thereafter, many studies have shown *GNAS* pathogenic variants occurring frequently in sporadic intramuscular myxomas [10, 18–22]. Additionally, *GNAS* pathogenic variants are absent in low-grade myxofibrosarcoma, which can be used as a malignant differential diagnosis to intramuscular myxomas [23, 24]. A precise diagnosis between these two is essential for the correct treatment; low-grade myxofibrosarcomas commonly recur and require wide excisions, whereas a more precise or marginal excision is sufficient to treat intramuscular myxomas [24, 25].

Intramuscular myxomas are challenging to diagnose, especially on core needle biopsies [26]. Also, they have a broad differential diagnosis at a morphological level and no clear karyotypic diagnosis [22, 27]. Additionally, there are many different types of *GNAS* pathogenic variants found in intramuscular myxomas, located on either codon 201, in exon 8, or codon 227, in exon 9. *GNAS* also has a low range of pathogenic allele frequencies in intramuscular myxomas, ranging between ∼ 5–30%. The most common pathogenic variants are p.R201H (c.602G>A) and p.R201C (c.601C>T), making up approximately 80%–90% of all cases [10, 19, 20, 22]. This has influenced the design of some diagnostic methods to only detect common pathogenic variants, and not the rarer ones [28, 29]. Depending on the method, *GNAS* pathogenic variants were found in ∼30–80% of intramuscular myxomas [20–22].

The gold standard for sensitive screening for genes that can predispose a patient to particular cancer types, such as pathogenic variants in *GNAS*, was PCR amplification, followed by sequencing, e.g., Sanger sequencing (also known as dideoxy chain termination method) of the amplified product [30–34]. However, in the last two decades, next-generation sequencing (NGS, also known as high throughput sequencing and massive parallel sequencing) has become common practice in cancer genetic screening [35]. Methods such as whole genome sequencing, exome sequencing, and transcriptome sequencing can provide more sensitive and accurate results. The main difference between Sanger sequencing and NGS is the sequencing volume. The Sanger methodology can only sequence a single DNA fragment at a time, whereas NGS simultaneously sequences millions of fragments per run. Thus, NGS results in hundreds to thousands of genes being sequenced simultaneously. Additionally, NGS can start with relatively low input DNA compared to that of Sanger methodology and provides enhanced detection capabilities to detect novel or rare variants through deep sequencing. Comparing the results obtained from NGS and Sanger sequencing there is close to 100% concordance between these methodologies [36–38]. NGS-based gene panels have also been developed to target specific genes or pathogenic variants (hotspots) that are associated to a particular cancer type [39–41]. These panels are nowadays used in clinical cancer genetics, providing accurate information on oncogenic drivers and actionable genetic alterations for a variety of genes [39–41]. The commercially available Ion AmpliSeq Cancer Hotspot Panel v2 (ThermoFisher Scientific, Waltham, MA, United States) covers approximately 2,800 pathogenic variants which are found in the Catalogue Of Somatic Mutations In Cancer (COSMIC) [42]. The pathogenic variants detected by Ion AmpliSeq Cancer Hotspot Panel v2 are from 50 oncogenes and tumor suppressor genes, including those at codons 201 and 227 of *GNAS*.

In the present study, we cytogenetically analyzed intramuscular myxomas and compared a PCR based/Sanger sequencing methodology with the above-mentioned panel for the detection of *GNAS* pathogenic variants in codons 201 and 227.

## Materials and methods

### Patients

Our materials consisted of 22 intramuscular myxomas (Table 1) and all samples were collected at the Norwegian Radium Hospital between 2015 and 2023. The patients were 17 females and five males between the ages of 39 and 80. The study was approved by the Regional Committees for Medical Research Ethics South East Norway.

**TABLE 1:** Clinicopathological data, karyotypes, and status of pathogenic variants of the *GNAS* gene on the intramuscular myxomas.

Case	Sex/Age	Location	Karyotypes	Amino acid sequence change^a^	Nucleotide sequence change^b^	Allele frequency^c^
1	F/55	Thigh	47,XX,+8 [2]/46,XX [24]	p.R201H	c.602G>A	7.80%
2	F/45	Thigh	46,XX,t (6; 13) (p23; q14)[3]/46,XX [22]	p.R201H	c.602G>A	11.40%
3	F/66	Upper arm	46,XX,inv (12) (q13q21)[2]/46,XX [23]	p.R201H	c.602G>A	8.20%
4	F/49	Thigh	46,XX,t (10; 22) (q24; q13)[3]/46,XX [22]	p.R201H	c.602G>A	7.90%
5	F/39	Buttock	46,XX	p.R201H	c.602G>A	13.50%
6	M/74	Thigh	46–47,X,-Y,+X,add (8) (p?11),+9 [cp5]/46,XY [15]	p.R201H	c.602G>A	16.70%
7	F/78	Back	49,XX,+X,+7,+8 [5]/46,XX [5]	p.R201H	c.602G>A	4.30%
8	F/58	Thigh	47,XX,+7 [3]/46,XX [10]	p.R201H	c.602G>A	5.20%
9	F/52	Thigh	46,XX	p.R201H	c.602G>A	33%
10	F/56	Thigh	46,XX	p.R201C	c.601C>T	7.40%
11	F/54	Thigh	46,XX	p.R201C	c.601C>T	28%
12	M/64	Thigh	46,XY	p.R201C	c.601C>T	21.90%
13	F/46	Thigh	46,XX	p.R201C	c.601C>T	10.10%
14	F/58	Upper arm	46,XX	p.R201C	c.601C>T	13.70%
15	F/67	Buttock	46,XX	p.R201S	c.601C>A	15.90%
16	F/71	Hip	50,XX,+7,+8,+8,+20 [8]/46,XX [2]	p.R201S	c.601C>A	7%
17	F/80	Leg	47–48,XX,+7 [cp3]/48,XX,+7,+8 [4]/46,X,-X,+5 [4]/46,XX [22]	p.R201S	c.601C>A	5.30%
18	M/66	Thigh	46,XY	p.R201G	c.601C>G	18.40%
19	M/71	Thigh	46,XY,add (15) (p11∼13)[2]/46,XY [8]	p.Q227E	c.679C>G	11.60%
20	M/69	Thigh	48,XY,+7,+9 [5]/46,XY [20]	No pathogenic variant	—	—
21	F/58	Thigh	46,XX	No pathogenic variant	—	—
22	F/79	Back	46,XX	No pathogenic variant	—	—

^a^ Based on GNAS reference sequence NP_000507.1.

^b^Based on *GNAS* reference sequence: NM_000516.7.

^c^ Allele frequency obtained with Ion torrent/Ion AmpliSeq Cancer Hotspot Panel v2. It is defined as the ratio between allele coverage and total coverage.

### G-banding and karyotyping

A representative tumor area was investigated cytogenetically as previously described [27, 43]. Material for cytogenetic examination was available from all 22 samples. After mechanical and enzymatic disaggregation of the tissue sample, the resulting cells were short-term cultured, harvested, and processed for cytogenetic examination. To obtain G-banding of chromosomes, Wright’s stain was used (Sigma Aldrich; St Louis, MO, United States). The subsequent cytogenetic analysis and karyotype description followed the recommendations of the International System for Human Cytogenomic Nomenclature (ISCN) 2020 guidelines [44].

### DNA extraction from tumor and FFPE tissue samples

Frozen (−80°C) tumor tissue adjacent to that used for cytogenetic analysis and histologic examination was available for 21 myxomas (Table 1; Supplementary Table S1) and formalin-fixed, paraffin-embedded (FFPE) sample was used for case 14 (Supplementary Table S1). DNA was extracted from the frozen tumor samples, using the Maxwell RSC Tissue DNA Kit and the Maxwell RSC Instrument (Promega, Madison, WI, United States). For FFPE sample, DNA was extracted using the QIAamp DNA FFPE Tissue Kit (Qiagen, Hilden, Germany). The DNA concentration was then estimated for each sample, using the QuantiFluor ONE dsDNA System using Quantus™ Fluorometer (Promega).

### Sequencing of *GNAS* pathogenic variants using Sanger sequencing

The primers used for PCR amplification and Sanger sequencing are listed in Table 2. The BigDye Direct Cycle Sequencing Kit was used for PCR/cycle (Sanger) sequencing according to the company’s recommendations (ThermoFisher Scientific, Waltham, MA, United States). The primer combination M13For-GNASint7-F1 and M13Rev-GNASint8-R1 was used for amplification and sequencing of exon 8 of *GNAS* to detect possible pathogenic variants at the codon 201 (p.R201) (Table 2). The primer combination M13For-GNASint8-F1 and M13Rev-GNASint9-R1 was used for amplification and sequencing of exon 9 of *GNAS* to detect possible pathogenic variants at codon 227 (p.Q227) (Table 2). Sequencing was run on the Applied Biosystems SeqStudio Genetic Analyzer system (ThermoFisher Scientific). For analysis of sequence data, the basic local alignment search tool (BLAST) software^1^ was used [45]. The obtained sequences were aligned against the *GNAS* reference sequences NM_000516.7 and NG_016194.2 corresponding to GNAS complex locus (*GNAS*) transcript variant 1 and RefSeqGene on chromosome 20, respectively. Codons 201 (p.R201) and 227 (p.Q227) are based on the NCBI reference sequence NP_000507.1 which corresponds to protein GNAS isoform GNASL.

**TABLE 2:** Designation, sequence (5′->3′), and position in reference sequence of the forward (F) and reverse (R) primers used for BigDye^TM^ Direct Cycle Sequencing of exons 8 and 9 of the *GNAS* gene.

Primer designation	Sequence (5′->3′)	Reference sequence: Position
**M13For-**GNASint7-F1	***TGTAAAACGACGGCCAGT*** ACT​GTT​TCG​GTT​GGC​TTT​GGT​GA	NG_016194.2: 74,561–74583
**M13Rev-**GNASint8-R1	***CAGGAAACAGCTATGACC*** CAG​AGG​GAC​TGG​GGT​GAA​TGT​CA	NG_016194.2: 74,752–74730
**M13For-**GNASint8-F1	***TGTAAAACGACGGCCAGT*** TGA​CAT​TCA​CCC​CAG​TCC​CTC​TG	NG_016194.2: 74,730–74752
**M13Rev-**GNASint9-R1	**CAGGAAACAGCTATGACC** AGC​GAC​CCT​GAT​CCC​TAA​CAA​CAC	NG_016194.2: 74,910–74887

The forward primers have the **M13** (highlighted in bold) forward primer sequence TGTAAAACGACGGCCAGT at their 5′-end. The reverse primers had the M13 reverse primer sequence CAGGAAACAGCTATGACC at their 5′-end.

### Sequencing of *GNAS* pathogenic variants using Ion AmpliSeq Cancer Hotspot Panel v2

The Ion Ampliseq™ Cancer Hotspot Panel v2 Chef-Ready Kit was used to detect *GNAS* pathogenic variants following the company’s recommendations (ThermoFisher Scientific). DNA samples were diluted to between 0.4 and 1.6 ng/μL or used as is when the sample concentration was below 0.6 ng/μL. All runs on the Ion Chief and Ion torrent were created in TorrenSuite™ (ThermoFisher Scientific). Libraries were prepared using Ion Ampliseq™ Cancer Hotspot Research Panel v2 primers and by using the Ion AmpliSeq™ Kit for Chef DL8 and were run using recommended setting from the company (ThermoFisher Scientific). All samples were templated via the automated template preparation protocol Ion 540™ Kit—Chef (ThermoFisher Scientific), which was then sequenced on the Ion GeneStudio S5 following the company’s recommendations (ThermoFisher Scientific).

## Results

### Cytogenetics

**FIGURE 1:**
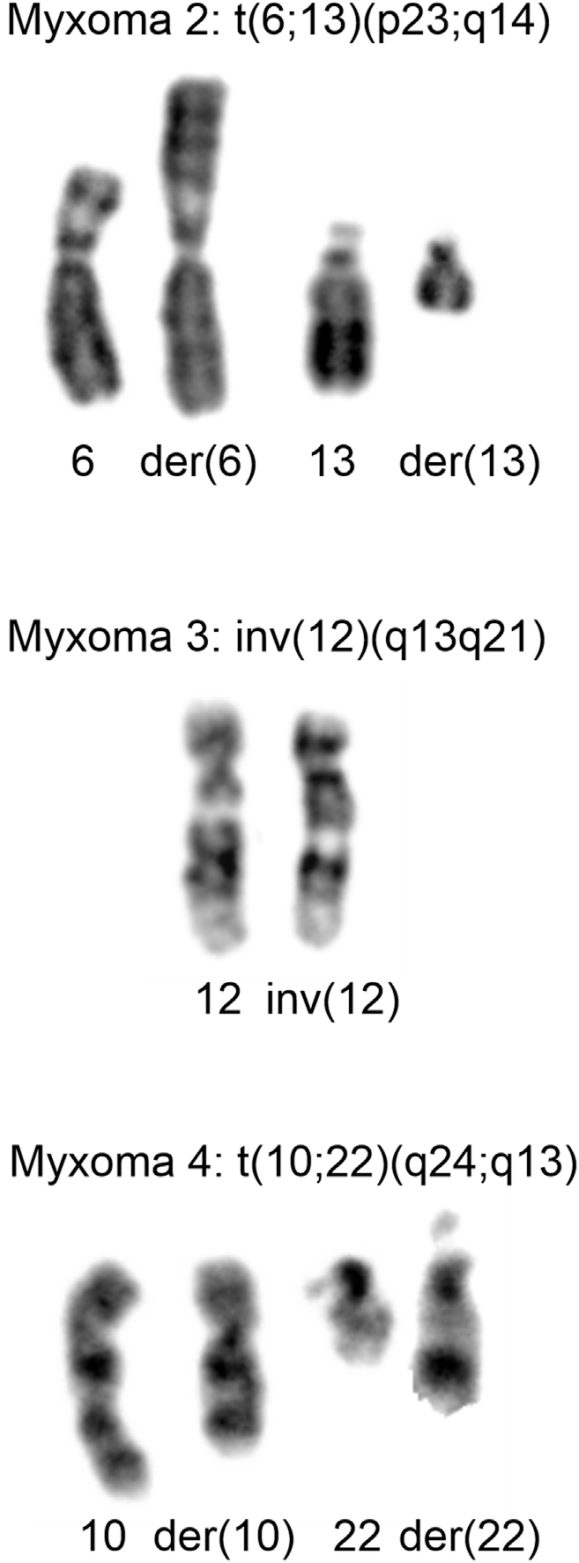
Cytogenetic analysis of myxomas 2, 3, and 4. Partial Karyotypes showing the abnormal chromosomes together with the corresponding normal chromosome homologs. For myxoma 2, der (6) and der (13), together with chromosomes 6 and 13 are shown. For myxoma 3, inv (12) together with normal 12 are shown. For myxoma 4, der (10) and der (22), together with chromosomes 10 and 22 are shown.

The cytogenetic data are presented in Table 1. Normal karyotypes were found in 11 intramuscular myxomas and were abnormal in 11 tumors. Six abnormal karyotypes had numerical chromosomal aberrations, involving one or more of chromosomes X, 7, 8, 9, 20, and four tumors had structural aberration, and one had both numerical and structural aberrations (Table 1). Gains of chromosome 7 were the most frequent abnormalities found in five myxomas, followed by gains of chromosome 8, which were found in four tumors. No recurrent structural aberration was found (Table 1). The structural aberrations were t (6; 13) (p23; q14), inv (12) (q13q21), t (10; 22) (q24; q13), add (15) (p11∼13), and add (8) (p?11) (Figure 1; Table 1).

### Sanger sequencing

**FIGURE 2:**
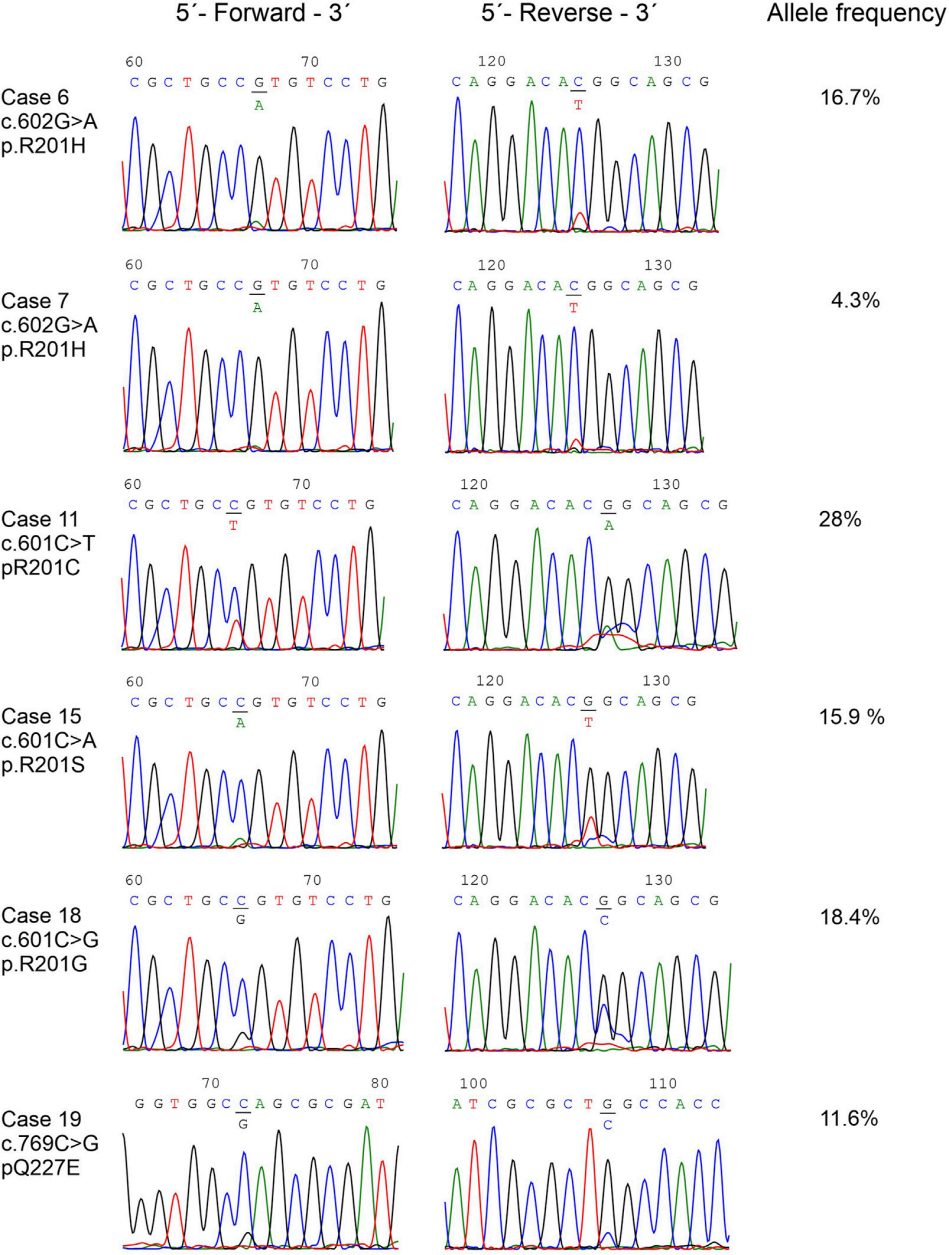
The detected *GNAS* pathogenic variants. Partial chromatograms showing the *GNAS* pathogenic variants in cases 10, 11, 13, 16, 18, and 19. Chromatograms from both forward and reverse sequencing are shown. The frequencies of mutated alleles, found with the Ion Ampliseq™ Cancer Hotspot Panel v2, are also shown.

DNA was successfully extracted from all tumor samples. Pathogenic variants in the *GNAS* gene were detected in 19 out of 22 myxomas (86%) (Supplementary Table S1). The detected pathogenic variants were p.R201H in nine cases which were found in seven myxomas with abnormal karyotypes and two with a normal karyotype, p.R201C in five cases all of them with normal karyotypes, p.R201S in three cases which two of them had abnormal karyotype and one had a normal karyotype, p.R201G in one case with a normal karyotype, and p.Q227E in one case with an abnormal karyotype (Table 1; Figure 2).

### Ion AmpliSeq Cancer Hotspot Panel v2

The Ion torrent results were the same as that of Sanger sequencing (Supplementary Table S1) and are therefore both represented in Table 1 for each case. Pathogenic variants in the *GNAS* gene were detected in the same 19 samples in which were detected by Sanger sequencing. Ion torrent also provided the allele frequencies of the mutated alleles ranging from 4.3%–33%, with an average of 13% (Table 1).

## Discussion

Over 40,000 new GNAS p.R201 pathogenic variant cases were reported in 2021 in the United States alone, illustrating the high prevalence of this genetic abnormality and importance of detecting this genetic abnormality [15]. Diagnosing myxoma is difficult as there are no useful immunohistochemical markers to distinguish intramuscular myxomas from low-grade myxofibrosarcomas, both have an abundant myxoid matrix, and both may express the CD34 molecule [22, 46, 47]. Detection of acquired genetic abnormalities in combination with histopathology, can be a powerful diagnostic tool to help distinguish between morphologically similar neoplasms, such as intramuscular myxomas and low-grade myxofibrosarcomas [18, 23, 24]. Morphologically, myxofibrosacomas have more prominent blood vessels and nuclear atypia, but cellular myxomas share similar features, so morphology alone is not always sufficient to make a definite diagnosis, especially in small biopsies. Additionally, small biopsies may also create issues with pathogenic variants detection which can be hampered by low frequency of pathogenic variants alleles, i.e., sequences of mutated alleles “diluted” by wild-type sequences. For pathogenic variant detection it is recommended to analyses at least 50% lesioned tissue. This may require painstaking and time-consuming micro-dissection of the relevant tissue and can be difficult to achieve in samples from needle biopsies [18, 21]. It has been suggested that a minimum of 10% tumor cell fraction is required to detect *GNAS* pathogenic variants [22], but the tumor cell fraction may have not been estimated accurately [48, 49]. This is especially difficult in intramuscular myxoma tumors, even when using fully excised tumors, as there is low amounts of DNA available for extraction and a generally low level of pathogenic variants which could easily become diluted by wild-type sequences [18].

Another complication is that there are no pathognomonic chromosomal aberrations for intermuscular myxomas [27]. The abnormal karyotypes, which were found in 11 out of 22 myxomas (Table 1), confirmed the result and conclusion of our previous study [27]. Abnormalities of chromosomes 7 and 8, both mainly trisomy, were the most common aberrations. These abnormalities lack specificity and are found in a variety of different neoplasms [50–55] and trisomy 7 is also seen in non-neoplastic lesions [27, 56] Furthermore, the observed structural chromosomal aberrations were not recurrent (Table 1; [27]). Even though “acquisition of clonal chromosome aberrations is an integral part of the disease process” in intermuscular myxomas, the cytogenetic information cannot be used for the diagnosis of myxomas (Table 1, [27]).

A useful tool for the diagnosis of intramuscular myxomas is that intramuscular myxomas often carry a somatic *GNAS* pathogenic variants. Since *GNAS* pathogenic variants are absent in low-grade myxofibrosarcoma, detection of these pathogenic variants can be a useful differential diagnosis [10, 18–24]. Detecting pathogenic variants within the *GNAS* gene in intramuscular myxoma can be difficult due to the poor cellularity of the tumor and the low frequency of mutated alleles, which was found to be between 5% and 30%, [3, 18, 20–22]. A further complication for *GNAS* pathogenic variant detection is that PCR-based methods can produce varying results. Okamato et al. (2000) detected *GNAS* pathogenic variants in five out of six (83%) intramuscular myxomas (60% p.R201H and 40% p.R201C) using PCR methodology together with single strand conformation polymorphism [10, 57]. Delaney et al. (2009) detected pathogenic variants in eight out of 28 (29%) intramuscular myxomas using PCR followed by pathogenic variant-specific restriction enzyme digestion [18]. However, they found *GNAS* pathogenic variants in 17 out of 28 (61%) intramuscular myxomas with COLD-PCR followed by pathogenic variant-specific restriction enzyme digestion [18]. Walther et al. (2014) detected pathogenic variants in 23 out of 63 (36%) intramuscular myxomas (52% p.R201C and 48% p.R201H) using PCR and direct sequencing [19]. In the present work, we detected *GNAS* pathogenic variants in 19 out of 22 samples (86%) using direct cycle Sanger sequencing with primers tailed on their 5′-end with M13 universal primer sequences (Table 2). We detected a total of five different pathogenic variants: p.R201H (47.3%), p.R201C (26.3%), p.R201S (16%), p.Q227E (5.2%), and the rare pathogenic variant p.R201G (5.2%) (Table 1; Figure 2). The p.R201G pathogenic variants has so far only been reported in one patient with McCune-Albright Syndrome over 20 years ago [58]. One complication when using Sanger sequencing is that it can be hard to detect allele frequencies below 15%. Intramuscular myxomas tumors have low allele frequencies of GNAS pathogenic variants and in our investigation, we found an average allele frequency of 13% (Table 1). To combat this a positive result from both bidirectional Sanger sequencing is mandatory, and reruns are used to confirm if only one direction is positive. Additionally, Sanger sequencing cannot accurately determine the allele frequency and is only used as a qualitative method (Yes/No) whereas NGS can be used as both qualitative and quantitative (Yes/No and frequency of various alleles) (Table 1; Figure 2).

NGS has also been used to detect *GNAS* pathogenic variants in intramuscular myxomas [20–22]. Sunitsch et al. (2018) detected *GNAS* pathogenic variants in 12 out of 13 (92%) specimens (four cases with p.R201C, six cases with p.R201H and two cases with p.Q227E) using Ion Ampliseq primer panel and Ion torrent sequencing [21]. Bekers et al. (2019) detected *GNAS* pathogenic variants in 16 out of 28 (57%) samples with frequency of the mutant allele between 5% and 27% using single molecule tagged molecular inversion probe assay and Illumina NextSeq sequencing [20]. In addition to p.R201H and p.R201C, which were detected in 44% and 31% of the samples respectively, four other *GNAS* pathogenic variants were found in intramuscular myxomas; p.R201S, p.R201L, p.R201P and p.Q227R [20]. Libbrecht et al. (2019) detected eight out of nine (89%) intramuscular myxomas to have a *GNAS* pathogenic variants (62.5% p.R201H and 37.5% p. R201C) with frequency of the mutant allele ranging between 5% and 28% using TruSight Tumor 26 panel [22]. In the present study, we detected five different *GNAS* pathogenic variants in 19 out of 22 samples 83%, with frequency of the mutated allele to ranging between 4.3% and 28% (Table 1) using the Ion Ampliseq™ Cancer Hotspot Panel v2. Our data obtained with the NGS panel were similar to the above-mentioned studies. We also found that results obtained from the NGS panel were identical to those obtained by direct cycle Sanger sequencing, indicating that the latter methodology is a highly sensitive technique to detect *GNAS* pathogenic variants. The present study (Table 1) together with the studies published by Bekers et al. (2019) and Sunitsch et al. (2019) showed that approximately 23% of myxomas with *GNAS* pathogenic variants carried variants in other locations other than the “hotspot” variants p.R201C and p.R201H. Thus, assays targeting only these pathogenic variants, p.R201C and p.R201H, such as pyrosequencing, quantitative real-time PCR, and digital PCR should be used with caution for the detection of *GNAS* pathogenic variants in intramuscular myxomas [20, 21, 59, 60].

Even though cytogenetic information cannot be used for the diagnosis of myxomas, we found an association between karyotypes and specific *GNAS* pathogenic variant. The pathogenic variant p.R201H was detected in seven tumors carrying abnormal karyotype and in only two tumors with normal karyotype. The detected p.R201C pathogenic variants were found in five tumors with only normal karyotype (Table 1). To the best of our knowledge, this association between cytogenetic and molecular aberrations in intramuscular myxomas is reported for the first time in the present study. Although the number of cases in this report make up to few to draw any conclusions, our data may imply functional variations between the different pathogenic variants. Very little is known about possible functional differences between *GNAS* pathogenic variants. All constitutive pathogenic variants activate Gsα adenylate cyclase with overproduction of cyclic adenosine monophosphate (cAMP) and are therefore often treated as one and the same [61–63]. *GNAS* pathogenic variants are associated with upregulation of Wnt/beta-catening signaling [64–66] and were found in many tumors [15, 67]. In murine models, either p.R201C or p. R201H resulted in fibrous dysplasia [64, 68]. In patients with fibrous dysplasia/McCune-Albright syndrome carrying p.R201H or p.R201C, no clear genotype-phenotype correlation was found [69, 70]. Additionally, in patients with fibrous dysplasia no association was found between age, site, size, specimen type and *GNAS* mutational status [71]. A pan-cancer cohort of patients from 1050 *GNAS* mutant tumors showed a heavy disposition to the p.R201 codon, with a classic gain-of-function mutation. Both p.R201H and p.R201C pathogenic variants were found to drive tumor cell growth both *in vitro* and *in vivo* [15]. *GNAS* was overexpressed in the LS174T cell line using a doxycycline-inducible promoter, and the resulting effect on clonogenic capacity was assessed [15]. In another study, the basal and maximal adenylyl cyclase activity (cAMP accumulation) dose response under isoproterenol stimulation was found to be higher in p.R201H than that of p.R201C [72]. However, the authors have suggested that the *GNAS* expression was not stringently controlled, and this result could be a technical issue. This warranted further experimentation with both pathogenic variants and wildtype alleles to further elucidate functional variation like our karyotype results suggest.

It has been suggested that there can be notable variability found in phenotypes, pathogenicity and oncogenic effects, based on the specific substitution at a single location [73–75]. For example, the pathogenic missense variants which alter codon 336 in the *GARS1* gene that results in different phenotypic expression in a range of genetic neuropathies [73]. Two pathogenic variants resulting in a missense modifying amino acid at codon 336 in the catalytic domain of *GARS1*, were found in two unrelated patients - one a female with infantile spinal muscular atrophy; and the second, a male with Charcot–Marie–Tooth disease type 2D [73]. Exchanges in amino acids that change the pH of the residue have also been linked to cancers [75]. White et al. (2017) found that the pathogenic variants resulting in a change from arginine residue to a histidine residue produced a rise in intracellular pH, which in turn, conferred these mutants with oncogenic effects [75]. This is the same change in amino acid residue we see in the amino acid pathogenic variant p.R201H which could suggest functional variations between the different pathogenic variants in *GNAS*.

In conclusion, acquired genomic abnormalities, both at chromosomal and molecular levels, are found in intramuscular myxomas. However, the detected chromosomal aberrations are not pathognomonic for myxomas since they lack specificity and are found in various neoplasms or even in non-neoplastic cells. In our experiment, we reliably detected somatic *GNAS* pathogenic variants at the molecular level. Together with H&E stain morphology and immunohistochemistry, we can reliably diagnose and detect the majority of intramuscular myxomas, which in turn leads to the appropriate treatment. The two methods used, i.e., direct cycle Sanger sequencing (BigDye Direct Cycle Sequencing Kit) and NGS panel (Ion AmpliSeq Cancer Hotspot Panel v2) gave identical results detected *GNAS* pathogenic variant in 83% of the examined myxomas. In 26% of the myxomas which carried *GNAS* pathogenic variants, both methods detected pathogenic variants other than the common “hotspot” of p.R201C and p.R201H. This shows that methods designed to detect only “hotspot” variants might give false negative results. Direct cycle Sanger sequencing is a quick, reliable, and relatively cheap method to detect the *GNAS* pathogenic variants, matching even the most cutting-edge of sequencing methods while only lacking the detection of frequency of mutant allele, not required for diagnostic purposes. Ultimately, this will allow laboratories that do not have the funding for expensive NGS methods to accurately diagnose *GNAS* pathogenic variants. This will find application not only in intramuscular myxomas but other tumor types as well.

## Data Availability

The original contributions presented in the study are included in the article/Supplementary Material, further inquiries can be directed to the corresponding author.

## Footnotes

1
https://blast.ncbi.nlm.nih.gov/Blast.cgi
